# Impact of Prefrontal Theta Burst Stimulation on Clinical Neuropsychological Tasks

**DOI:** 10.3389/fnins.2017.00462

**Published:** 2017-08-18

**Authors:** Raquel Viejo-Sobera, Diego Redolar-Ripoll, Mercè Boixadós, Marc Palaus, Antoni Valero-Cabré, Elena M. Marron

**Affiliations:** ^1^Cognitive NeuroLab, Faculty of Health Sciences, Universitat Oberta de Catalunya Barcelona, Spain; ^2^Laboratory for Neuropsychiatry and Neuromodulation, Department of Psychiatry, Massachusetts General Hospital, Harvard Medical School Boston, MA, United States; ^3^Cerebral Dynamics Plasticity and Rehabilitation Group, Frontlab, Institut du Cerveau et la Moelle Centre National de la Recherche Scientifique UMR 725, Institut National de la Santé et de la Recherche Médicale 1127 and UPMC Paris, France; ^4^Laboratory for Cerebral Dynamics Plasticity and Rehabilitation, Boston University School of Medicine Boston, MA, United States

**Keywords:** transcranial magnetic stimulation, non-invasive brain stimulation, stroop test, n-back, tower of hanoi, working memory, executive functions, dorsolateral prefrontal cortex

## Abstract

Theta burst stimulation (TBS) protocols hold high promise in neuropsychological rehabilitation. Nevertheless, their ability to either decrease (continuous, cTBS) or increase (intermittent, iTBS) cortical excitability in areas other than the primary motor cortex, and their consistency modulating human behaviors with clinically relevant tasks remain to be fully established. The behavioral effects of TBS over the dorsolateral prefrontal cortex (dlPFC) are particularly interesting given its involvement in working memory (WM) and executive functions (EF), often impaired following frontal brain damage. We aimed to explore the ability of cTBS and iTBS to modulate WM and EF in healthy individuals, assessed with clinical neuropsychological tests (Digits Backward, 3-back task, Stroop Test, and Tower of Hanoi). To this end, 36 participants were assessed using the four tests 1 week prior to stimulation and immediately following a single session of either cTBS, iTBS, or sham TBS, delivered to the left dlPFC. No significant differences were found across stimulation conditions in any of the clinical tasks. Nonetheless, in some of them, active stimulation induced significant pre/post performance modulations, which were not found for the sham condition. More specifically, sham stimulation yielded improvements in the 3-back task and the Color, Color-Word, and Interference Score of the Stroop Test, an effect likely caused by task practice. Both, iTBS and cTBS, produced improvements in Digits Backward and impairments in 3-back task accuracy. Moreover, iTBS increased Interference Score in the Stroop Test in spite of the improved word reading and impaired color naming, whereas cTBS decreased the time required to complete the Tower of Hanoi. Differing from TBS outcomes reported for cortico-spinal measures on the primary motor cortex, our analyses did not reveal any of the expected performance differences across stimulation protocols. However, if one considers independently pre/post differences for each individual outcome measure and task, either one or both of the active protocols appeared to modulate WM and EF. We critically discuss the value, potential explanations, and some plausible interpretations for this set of subtle impacts of left dlPFC TBS in humans.

## Introduction

Evidence-based guidelines for the therapeutic application of transcranial magnetic stimulation (TMS) in different disorders have recently been published (Lefaucheur et al., 2014). Promising results show improvements in cognitive performance in clinical populations with acquired brain damage, such as stroke (e.g., Shah et al., 2013) and traumatic brain injury (e.g., Ulam et al., 2015); psychiatric conditions such as major depression (e.g., Cheng et al., 2015) and schizophrenia (e.g., Barr et al., 2013); and neurodegenerative diseases, such as Parkinson's, Alzheimer's or mild cognitive impairment (e.g., Srovnalova et al., 2011, 2012; Devi et al., 2014; Drumond Marra et al., 2015). Nonetheless, its specific potential in cognitive rehabilitation, particularly when tested with clinically relevant neuropsychological assessment tools, remains elusive (Rossi and Rossini, 2004; Guse et al., 2010; Miniussi and Rossini, 2011; Anderkova and Rektorova, 2014).

It has been well-established that neural activity can be modulated using different repetitive TMS (rTMS) patterns (Thut and Pascual-Leone, 2010; Sandrini et al., 2011). A decade ago, patterned Theta Burst Stimulation (TBS) protocols, intermittent TBS (iTBS), and continuous TBS (cTBS), mimicking the protocols inducing LTP and LTD in animal models, were first implemented for non-invasive neuromodulation. Since then, they have become very popular due to their longer-lasting effects following short stimulation periods compared to most commonly employed classical rTMS paradigms (Huang et al., 2005; Suppa et al., 2016).

Both TBS protocols consist of 600 pulses delivered in brief trains (bursts) of three pulses with a frequency of 50 Hz administered every 200 ms (5 Hz). During cTBS, bursts are delivered without any interruption for a total of 40 s, while in iTBS, bursts are delivered during brief periods of 2 s (10 bursts) interleaved with 8 second-long stimulation-free intervals for a total duration of 190 s. The effects of iTBS and cTBS over the primary motor cortex assessed by means of cortico-spinally motor evoked potentials (MEPs) have thus far been estimated to induce changes of around 30% of MEP amplitude, lasting for up to 60 and 50 min, respectively (see Wischnewski and Schutter, 2015 for a review). It is worth noting that a high degree of interindividual variability in such cortico-spinal modulatory effects has been reported (Ridding and Ziemann, 2010; Hordacre et al., 2016), and the effects of TBS protocols over associative cortical areas have proven much less consistent (Grossheinrich et al., 2009; Brunoni and Vanderhasselt, 2014).

Extending the above-reported increases in MEP amplitudes (Huang et al., 2005) to cognitive domains, it would be generally assumed that increased cortical excitability, associated with high-frequency rTMS (>5 Hz) and iTBS, yields improvements in cognitive performance. In contrast, decreased excitability, often associated with low-frequency rTMS (≤1 Hz) and cTBS, should transiently degrade performance by inducing the so-called “virtual lesions” (Pascual-Leone et al., 1999; Lefaucheur et al., 2014). However, growing evidence suggests that both increases and decreases of cortical excitability may up- and down-regulate cognitive performance depending on the state of ongoing activity taking place before and during the stimulation, as well as the functional and structural connectivity patterns of the targeted area (Silvanto and Muggleton, 2008; Ruff et al., 2009; Pascual-Leone et al., 2012; Luber and Lisanby, 2014). The difficulty inferring TMS behavioral effects from the excitatory or inhibitory nature of the stimulation patterns becomes particularly important in highly interconnected areas involved multiple higher order processes such as the dorsolateral prefrontal cortex (dlPFC) (Duncan and Owen, 2000). Regardless, iTBS and cTBS continue to be applied to enhance and decrease excitability, with the aim of improving and impairing behavioral performance, respectively (e.g., Hoy et al., 2016; Christov-Moore et al., 2017).

The modulation of critical areas for cognitive processes such as working memory (WM) and executive functions (EF), frequently hindered in patients with diverse neurological conditions (e.g., Owen et al., 1990; Levy et al., 2002; Silver et al., 2003), is particularly relevant for rTMS therapeutic applications in rehabilitation. A common target related to both processes is the dlPFC (Miller and Cohen, 2001; Barbey et al., 2013). Hence, not surprisingly, studies aiming at cognitive enhancement or rehabilitation using non-invasive brain stimulation have frequently targeted this region (Pascual-Leone et al., 2012; Anderkova and Rektorova, 2014). The impact of TBS protocols on the dlPFC has been extensively tested using finely titrated computer-based tasks, ensuring that moderate excitability changes might result in significant behavioral modulations, whereas the use of ecological clinical tasks measuring different components of WM and EF remains scarce (Pascual-Leone et al., 2012). Better understanding the effects of TBS on cortical regions, other than on cortico-spinal systems, and accurately estimating its ability to influence behavioral performance in clinical tests is paramount in taking advantage of the promising capabilities of TBS in cognitive rehabilitation.

Here we aimed to explore the effects of cTBS and iTBS protocols applied over dlPFC on the performance of four commonly used clinical neuropsychological tests evaluating WM and EF: Digits Backward, Stroop Test, 3-back task and the Tower of Hanoi; all of them critically involving dlPFC activation as shown in neuroimaging and lesion studies (e.g., Goel and Grafman, 1995; Banich et al., 2000; Gerton et al., 2004; Owen et al., 2005). We initially predicted that active TBS but not sham protocols would have an impact on performance outcomes and also that the effects of cTBS and iTBS would be different. More specifically, we hypothesized that cTBS would be more likely to degrade performance while iTBS would improve it. Nevertheless, given the high variability of TBS effects on cortical excitability reported above, the dlPFC's rich widespread connectivity, and the fact that the four tasks do not necessarily assess exactly the same cognitive processes, we remained open to the observation of different effects and other subtle differences.

## Materials and methods

### Study participants

Thirty-six healthy volunteers (26 female) aged 18–57 years old (mean = 29.22; *SD* = 9.7) with college education took part in the study. All participants met the TMS safety criteria (Rossi et al., 2009). None of them wore eye makeup before stimulation to avoid local pain in the orbital area (Redolar-Ripoll et al., 2015) or had a previous or actual neurological disorder or a history of psychiatric illness, drug, or alcohol abuse. In order to navigate the TMS coil position during stimulation, a high-resolution structural MRI was obtained for each participant on a 1.5T scanner (Siemens Magnetom Essenza) at Hospital de Mollet, with an FSPGR-T1 3D sequence (slice thickness = 1 mm; TR = 500 ms; TE = 50 ms; matrix = 256 × 256; FOV = 240; 180 sagittal slices). The study was approved by the Ethics Committee of the Universitat Oberta de Catalunya. All participants gave written informed consent to participate in the study in accordance with the Declaration of Helsinki and received financial compensation for their participation.

### Procedure

**Figure 1:**
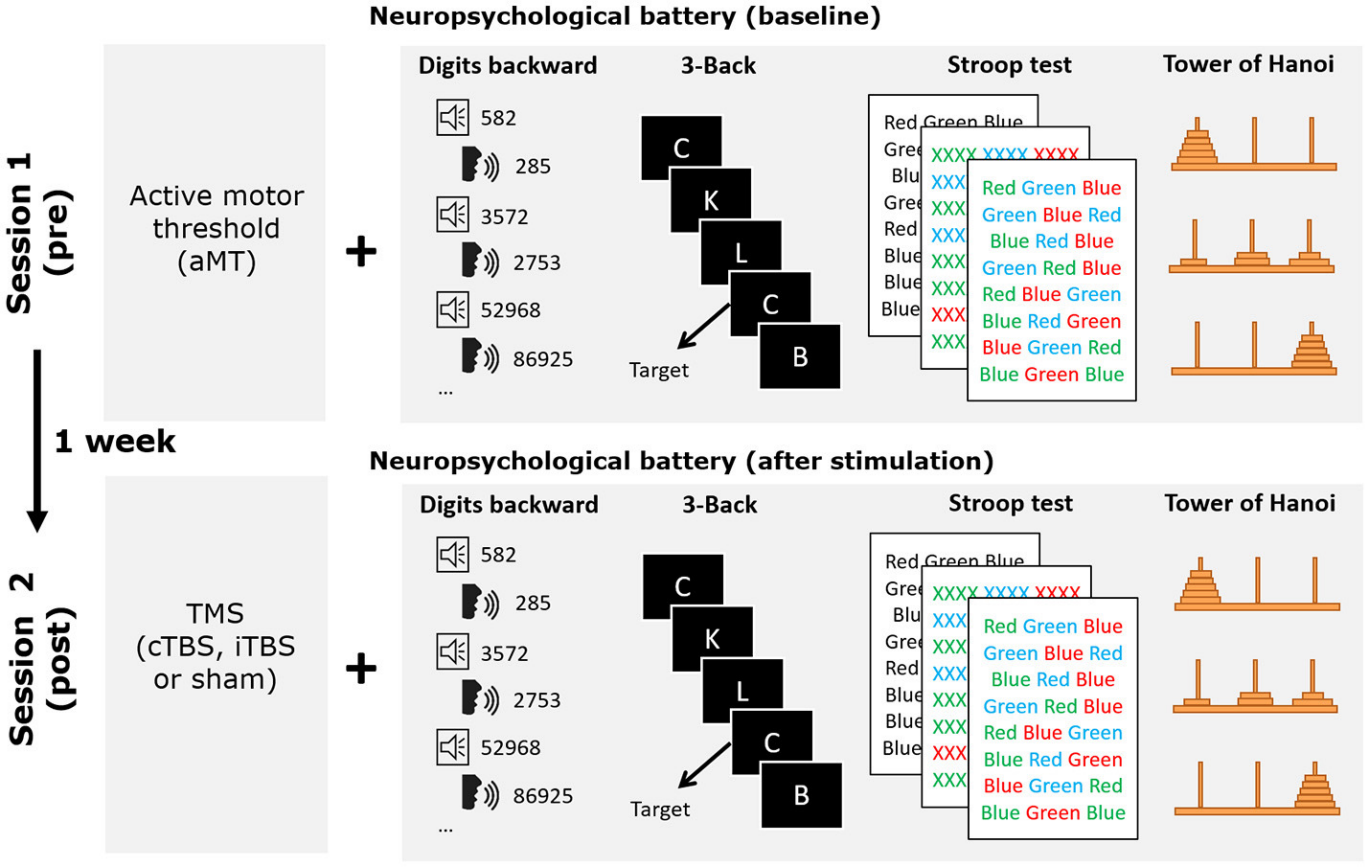
Schematic drawing representing the details of the experimental procedure followed by participants. During session 1 active motor threshold was determined before performing the neuropsychological battery of tests used in the study. One week thereafter, during session 2, the neuropsychological battery was performed again following one of the three TBS stimulation conditions (iTBS, cTBS or sham). Behavioral tasks were always administered in the same order.

The study consisted of a mixed factorial 3 × 2 design, with stimulation modality (iTBS, cTBS, sham) as a between-subjects factor, and time of assessment (baseline and after stimulation) as a within-subjects factor. At the expense of having to recruit more participants, we deliberately chose to compare the impact of TBS in three independent groups instead of using a crossover design performed by a single cohort. This decision was taken to avoid the effects of brain stimulation being masked by the effects of task practice by the repetition up to four times of the same evaluation tasks (baseline plus one time for each of the three rTMS conditions). Moreover, since offline rTMS crossover designs require counterbalancing the order of the tasks across participants and conditions, and adequate controlling for the effect of assessment time post stimulation on each task, an even higher number of participants would have been required. All participants carried out two sessions (session 1 and session 2; see Figure 1) 1 week apart at the facilities of the Cognitive NeuroLab in Barcelona. On each session participants performed the four clinical neuropsychological tasks once, following the same order (to limit variability generated by differences in the time elapsed since stimulation): Digits Backward, 3-back task, Stroop Color and Word Test, and the Tower of Hanoi. Sessions lasted ~30 min. Session 1 included the determination of the active motor threshold (aMT) and the assessment of baseline performance in the four tasks. In session 2, rTMS was administered immediately before participants performed these same four tasks sequentially, in the same order followed during baseline recordings. The likelihood of changes in the aMT after 1 week is very low given that its stability has been shown even across a 5 year period (Kimiskidis et al., 2004). Participants were randomly assigned to one of the three stimulation groups: iTBS, cTBS or sham (see TMS section for further details on the stimulation procedure). For the sham group, sham cTBS and sham iTBS were applied, respectively to half of the participant cohort. The results of all participants in the two sham modalities were combined for data analysis. All participants completed the Spanish versions of the Mini Mental State Examination and the Beck Depression Inventory before and after rTMS to control for any unexpected changes in mood or cognitive functioning.

### Neuropsychological assessment

The four evaluation tasks employed in the study were selected in order to assess participants' WM and EF. The Digits Backward test and the 3-back task are widely used for WM assessment since both involve the temporal storage and mental manipulation of information. Nonetheless, the 3-back task also requires the ongoing adjustment of the stored stimuli, incorporating new ones while rejecting old ones (Owen et al., 2005; Lezak et al., 2012). The Stroop Test and the Tower of Hanoi are considered measures of different components of the executive system. The Stroop effect (Stroop, 1935) is used to assess the interference control capacity, or the ability to inhibit automatic responses (Strauss et al., 2006), while the Tower of Hanoi is a measure of planning abilities, visuospatial memory, WM and response inhibition (Goel and Grafman, 1995; Miyake et al., 2000; Lezak et al., 2012). Digits Backward and 3-back task were computer-based versions, while Stroop Test and Tower of Hanoi were the classical, clinically used versions. For the computer-based tasks, stimuli presentation and response recording were controlled by stimulus presentation software (E-Prime 2.0, Psychology Software Tools) and displayed on a 17″ CRT screen (75 Hz). During the experiment, participants were comfortably seated at ~60 cm from the screen. The Stroop Test and the Tower of Hanoi were administered at the same desk where the computer was placed. The examination was carried out by an expert neuropsychologist. The whole assessment lasted for ~20 min.

#### Digits backward

We administered a computer-based version of the subtest included in the Wechsler Adult Intelligence Scale III (WAIS-III; Wechsler, 1999). In this version, digits were presented via audio recording in random order, paced at a frequency of 1/s. Participants had to verbally repeat the string in reverse order as previously heard while the examiner documented in writing their responses. Two series of digits of the same length (between 2 and 9 digits) were presented. The task was completed when participants failed two series of the same length. The maximum length of the string (span) and the number of series correctly completed were recorded.

#### 3-back task

This experimental task was based on the modified version (Salat et al., 2002) of a popular sequential letter task used in fMRI studies of WM (Braver et al., 1997; Cohen et al., 1997). Participants had to indicate whether the letter presented in the center of a computer screen was the same as the letter presented three positions before (3-back). Participants responded by pressing the key “J” of a keyboard using their right index finger when the letter was the same (target) or the key “K” with their right middle finger when the letter was different (non-target). All the letters of the alphabet, except vowels, were randomly presented. A total of 63 trials were performed, but the first three (non-target) were not included in the analyses. The number of target stimuli was 20 (i.e., ~32% of the total set). Letters were presented in white against a black background in Arial font size 30 (1.4 × 1–1.5 cm). Stimulus duration was 500 ms with a 2,500 ms interstimulus interval. Participants performed 20 trials of a practice block before the experiment. If they achieved <60% of correct responses, they were requested to repeat the practice block. The total duration of the task was ~4 min and 30 s, including practice. Both accuracy and reaction times were recorded.

#### Stroop color and word test

We administrated the standard Spanish version of the Stroop Test (Golden, 2001), consisting of three parts: Word Reading (WR), Color Naming (CN), and Color-Word (CW). In each part, participants were requested to read (WR) or name the color of the ink of each item (a series of X in CN and the name of a color non-coincident with the ink in CW) in a list of 100 elements. For each part, we recorded the number of elements correctly completed in 45 s and the Interference Score (IS). The IS, which is considered a measure of inhibitory control, was calculated from the other three scores [IS = CW − (WR^*^CN/WR+CN)] (for further information see Strauss et al., 2006, p. 477).

#### Tower of hanoi

This task consisted of a board with three pegs set in line (1st, 2nd, and 3rd), from left to right and five wooden disks of different sizes. At the beginning of the task, the 5 disks were all in the left peg with the largest on the bottom and the smallest on top. Participants were asked to move the discs to the 3rd peg on the right keeping the same configuration (increasing disk size from top to bottom) and respecting three rules: (1) to move only one disk at a time; (2) that disks which were not being moved remained on a peg; (3) not to place a larger disk on a smaller disk. We recorded the total time needed to complete the task and the number of movements performed.

### Transcranial magnetic stimulation procedures

A Magstim Super Rapid2 stimulator (Magstim Company Ltd., Whitland, U.K.) with a 70 mm figure of eight coil was used to stimulate cortical regions. To determine the aMT, the coil was placed tangentially over the participant's right primary motor cortex (M1), with the handle positioned 45° backwards with respect to the head's midline. The coil was repositioned until the hotspot of the left hand first dorsal interosseous (FDI) muscle was located. The aMT was defined as the minimum TMS intensity able to produce an FDI muscle twitch in 5 of 10 trials, during a sustained gentle isometric contraction (~20% of its maximum). The mean aMT value for the recruited group of participants was 57.4 ± 4.8% of the maximum stimulator output.

**Figure 2:**
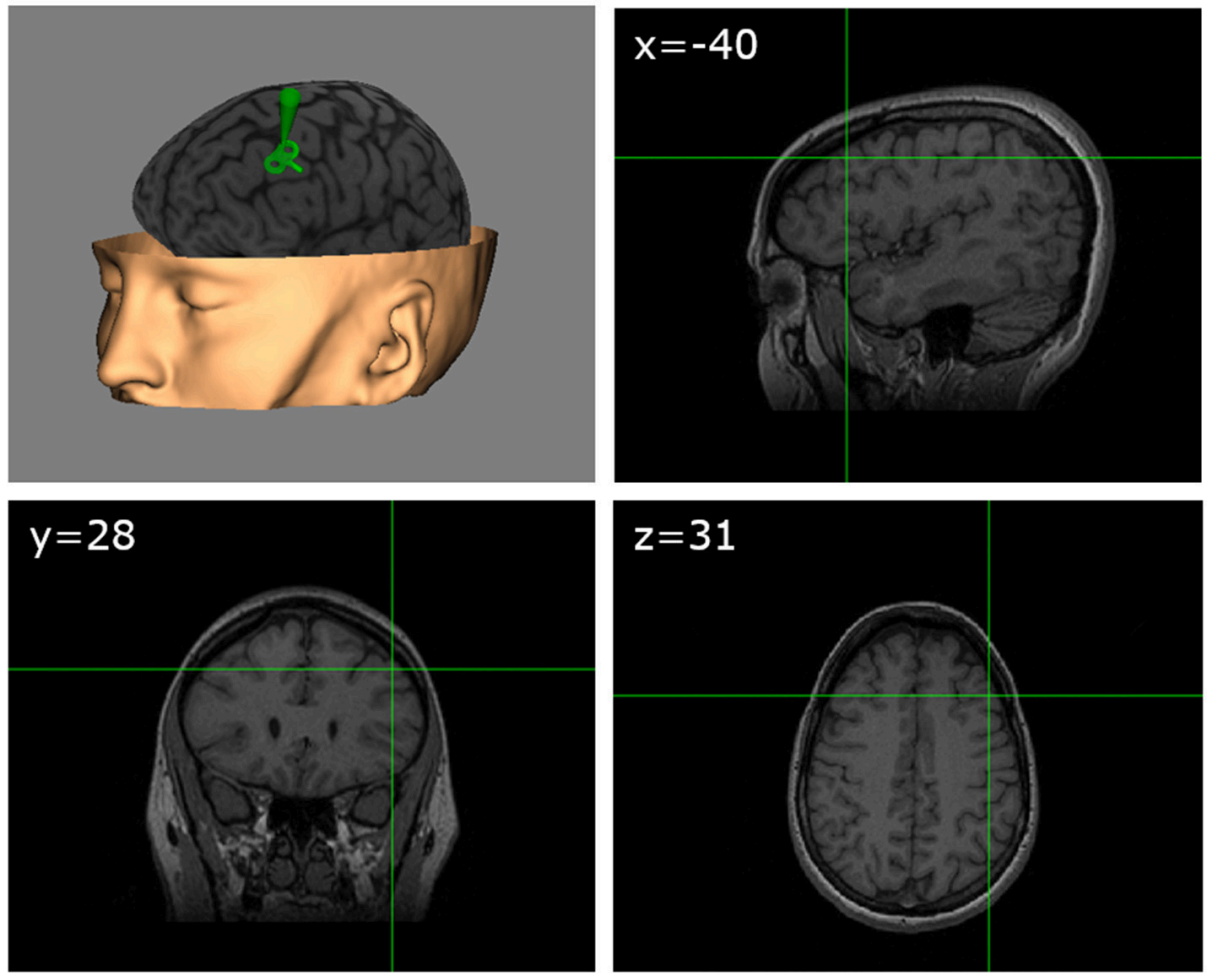
TMS target. Schematic figure showing on an MRI 3D reconstruction from a representative participant (with permission) the TMS targeted left dlPFC region identified according to standardized MNI coordinates (x = −40, y = 28, z = 31).

Our study targeted the left dlPFC. The site of stimulation in this area was determined based on previously published MNI coordinates (Gaudeau-Bosma et al., 2013) corresponding to BA9 (middle frontal gyrus, MNI: −40, 28, 31) in which decreases of BOLD activity had been observed during an n-back task following 10 sessions of rTMS stimulation. For each participant, the exact scalp location allowing the precise targeting of the coordinates of this area was anatomically determined through the individual MRI, using specialized frameless neuronavigation equipment and software (Brainsight, Rogue Research, Montreal, Canada; see Figure 2). Sham TMS stimulation (control condition) was delivered with the coil surface perpendicular to the scalp surface (90° angle), with the side of one of the coil loops in contact with the scalp vertex, hence directing the active magnetic field away from the brain. We deliberately chose this control procedure instead of active stimulation over a different cortical area (i.e., vertex) to avoid the confounding of nonspecific effects and/or specific effects influencing performance for any of the evaluation tasks. We also felt uneasy using sham stimulation with an active coil positioned on a 90° configuration on the dlPFC in view of reports suggesting significant influences of weak sham coil stimulation on frontal regions. In any case, given the *offline* design of our experiment, it is very unlikely that non-specific sensory effects related to TMS, such as the clicking noise or scalp-tapping sensations associated with each pulse could be held accountable for cognitive effects.

The TBS protocols applied delivered a total of 600 pulses organized in 50 Hz triplets repeated at 5 Hz, i.e., every 200 ms (Huang et al., 2011). The iTBS protocol repeats 2 s blocks of pulses interleaved with 8 s without stimulation 20 times, hence lasting for 3 min and 12 s. The cTBS protocol consists of a continuous repetition of bursts lasting 40 s. As indicated in the introduction, evidence obtained stimulating the M1 cortex has consistently demonstrated opposite effects on MEP amplitudes, with cTBS decreasing motor cortico-spinal excitability, and iTBS increasing it (Huang et al., 2005, 2009, 2011). Stimulus intensity was set at 80% of the individually estimated aMT following the common practice and the published international safety guidelines (Huang et al., 2005; Rossi et al., 2009; Suppa et al., 2016). Precise identification of left dlPFC target site, TMS coil positioning, and adequate targeting during stimulation was controlled with the above-mentioned frameless stereotaxic system (Brainsight, Rogue Research, Canada) equipped with an infrared tracking system (Polaris, Northern Digital, Waterloo, ON, Canada).

### Statistical analyses

**Figure 3:**
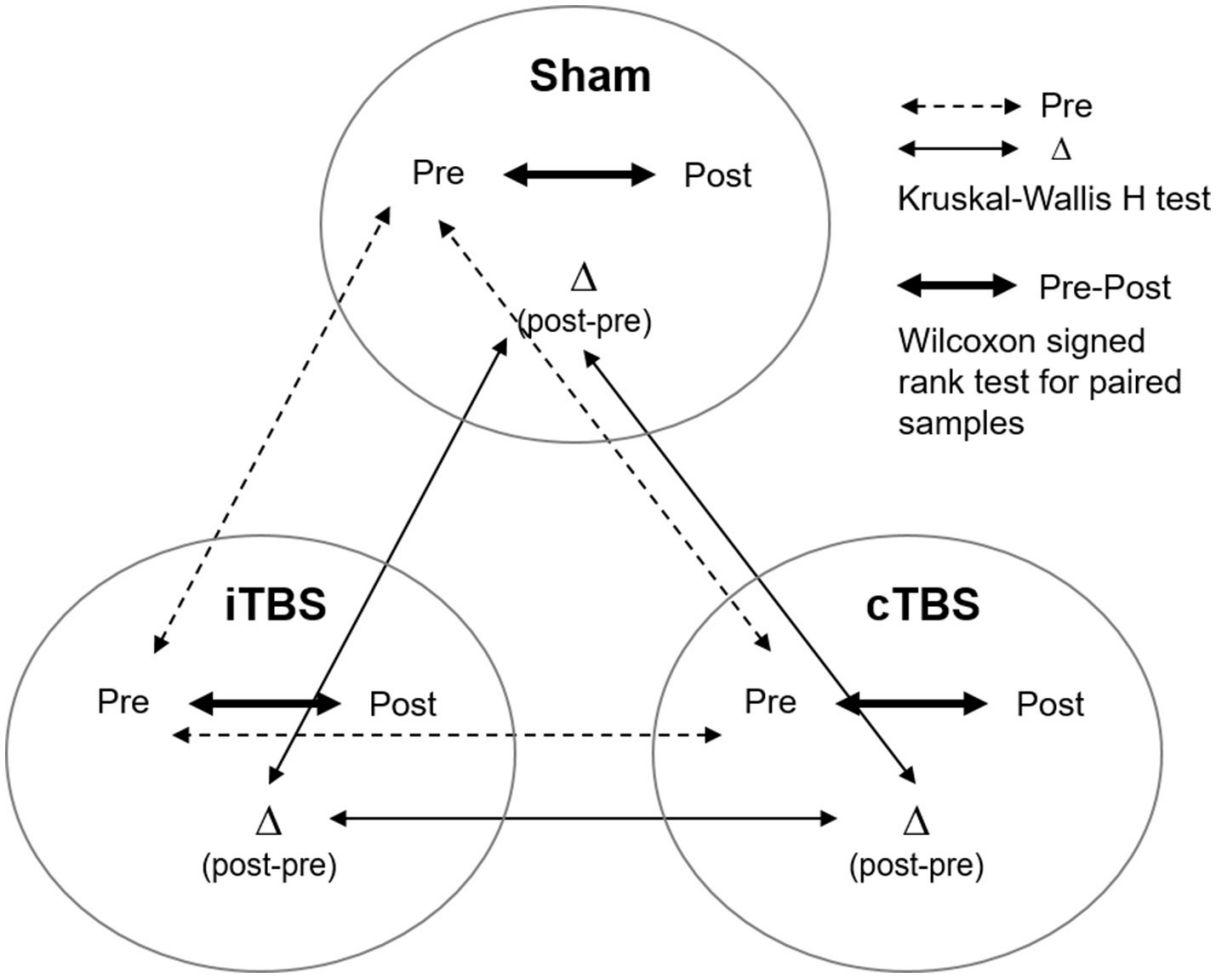
Representation of the different analyses performed on the current study to compare behavioral outcomes (pre, post stimulation, and delta post-pre stimulation change) across (Kruskal–Wallis *H*-test) and within (Wilcoxon signed rank test) stimulation conditions (i.e., sham, cTBS, and iTBS).

Analyses were performed with commercially available statistical software (SPSS software, version 23 and Stata Software version 15). The Shapiro–Wilk test was used to evaluate whether variables were normally distributed or not. Given the absence of normality for most of the scores (accuracy of the 3-back task, Digits Backward span, Stroop WR, CN, CW, number of movements and time in the Tower of Hanoi), we applied non-parametric statistical tests for all comparisons. A Kruskal–Wallis *H*-test was used to check for differences between groups at baseline (see Figure 3, dashed line). Pre- vs. post-TMS differences for each outcome measure were calculated by subtracting performance values estimated at baseline from those measured after TMS (Δ difference score, i.e., post-minus pre-TBS scores). The Kruskal–Wallis *H*-test for independent samples was used to compare the outcome measures (Δ difference score) across stimulation conditions (iTBS, cTBS, sham; Figure 3, thin line). In addition, in order to rule out the possibility of a type II error we conducted a nonparametric K-sample test on the equality of medians using the Fisher's exact sampling distribution and the chi-squared distribution. Wilcoxon signed rank test for paired samples was used to compare performance between baseline (pre) and after (post) TMS within each condition (Figure 3, thick line).

## Results

The comparison of the baseline measures between groups using the Kruskal–Wallis *H*-test (Figure 3, dashed line) showed no statistically significant differences for any variable [χ^2^_(2)_ < 5.03; ps > 0.081]. Given this result, the three groups were considered to have equivalent performance levels at baseline.

**Figure 4:**
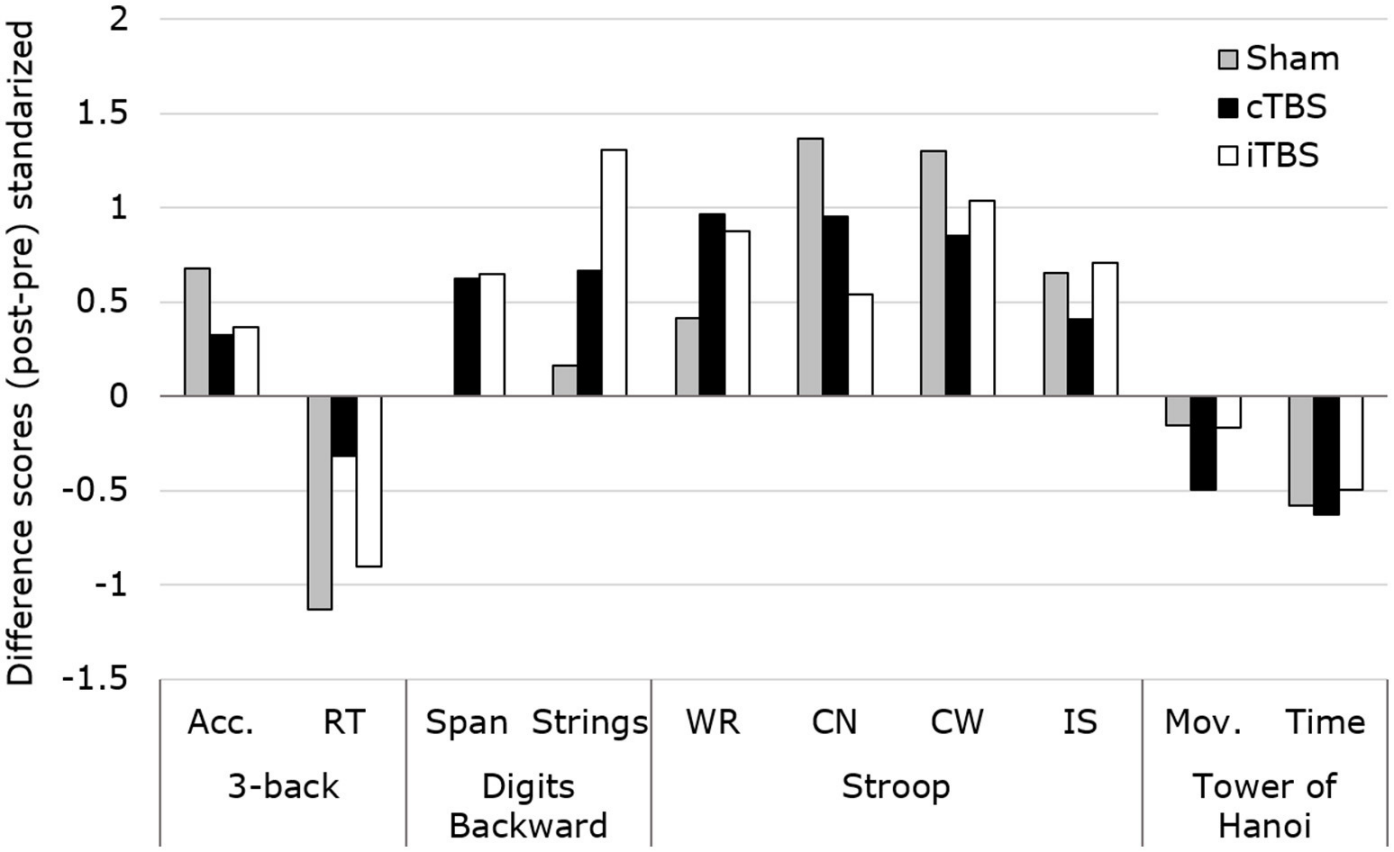
Representation of the standardized changes for each of the evaluated scores (mean/SD), across groups and tasks. The Kruskal–Wallis *H*-test revealed no statistically significant modulations of TBS patterns between conditions for any of the outcome measures. Acc., Accuracy; RT, reaction time; WR, Word Reading; CN, Color Naming; CW, Color-Word; IS, Interference Score; Mov., Number of movements.

The same Kruskal–Wallis *H*-test comparing the changes (Δ difference scores) in performance between the three groups (see Figure 3, thin line) revealed no significant differences in any of the 4 tasks of the study (asymptotic significance *p* = 0.07–0.86). These results were confirmed when assessing the equality of the medians (exact significance *p* = 0.14–1). This outcome does not support the expected predictions made for our study (see Figure 4).

Nonetheless, the Wilcoxon signed rank test did show statistically significant differences between pre- vs. post-stimulation in certain scores for the three stimulation conditions (Figure 3, thick line). In the next sections, we report these results for each task.

### Digits backward

The comparison of the digits backward span pre vs. post stimulation showed no significant differences for sham TBS (*Z* = −0.12; *p* = 0.903). In contrast, both iTBS (*Z* = −1.94; *p* = 0.052) and cTBS (*Z* = −1.89; *p* = 0.059) showed trends toward statistically significant performance increases in this task. This result suggests that iTBS and cTBS stimulation might have both equally influenced Digits Backward performance, without showing differences in effect direction as suggested by their ability to modulate cortico-spinal excitability in opposite directions.

**Figure 5:**
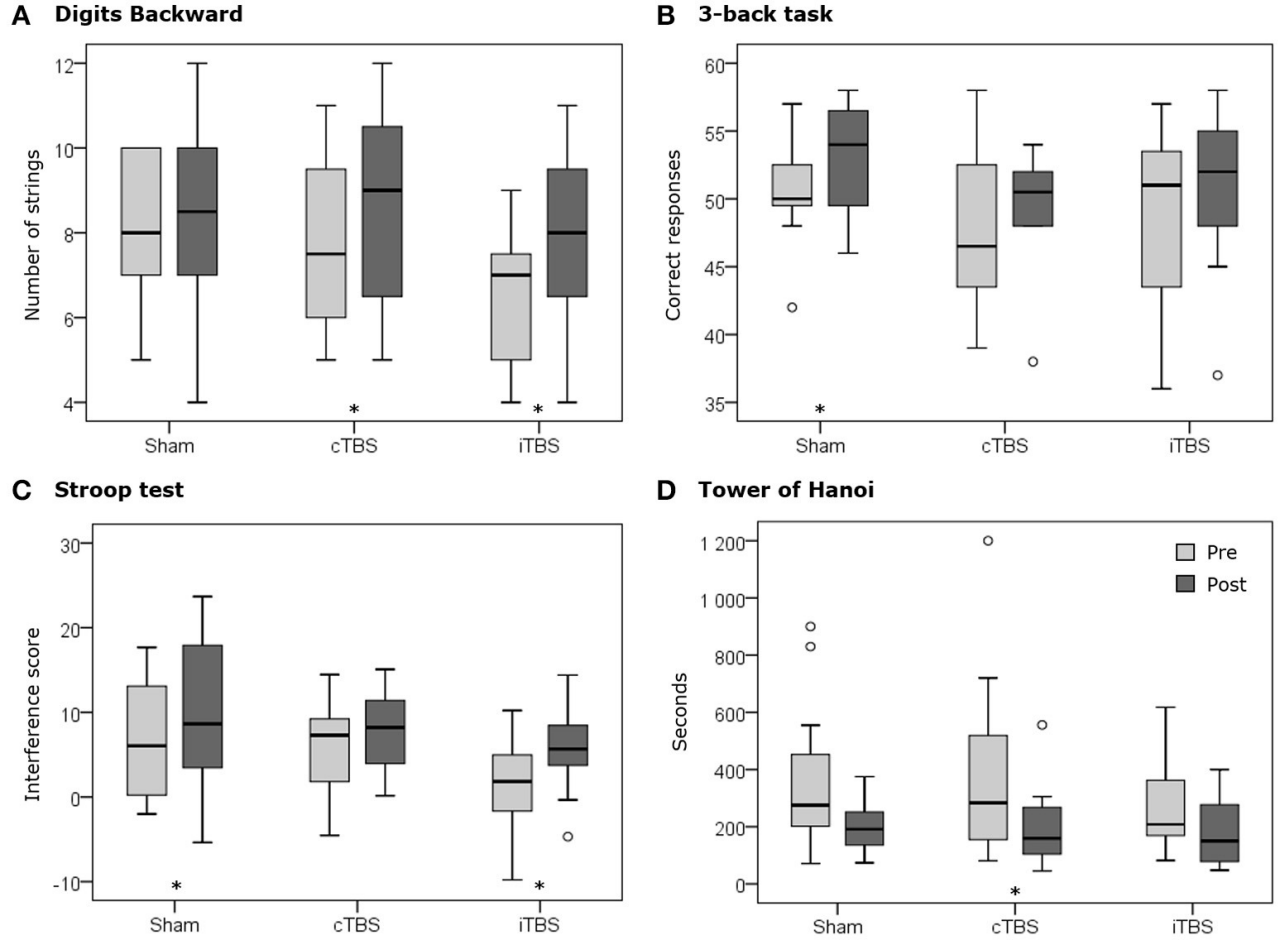
Boxplots representing the pre (pale gray) and post (dark gray) distributions of each stimulation condition (i.e., sham, cTBS and iTBS) for **(A)** the number of strings completed in the Digits Backward test, **(B)** the number of correct responses in the 3-back task, **(C)** the interference score of the Stroop test, and **(D)** the time to complete the Tower of Hanoi. The asterisks (^*^) represent statistically significant differences (*p* < 0.05) between pre- and post-TBS assessment in specific conditions and tests. Circles (°) represent outliers.

Regarding pre vs. post differences in the number of series correctly completed (strings), again sham stimulation showed no significant changes (*Z* = −0.57; *p* = 0.569). In contrast, both cTBS (*Z* = −2.07; *p* = 0.039), and iTBS (*Z* = −2.7; *p* = 0.007), yielded significantly higher levels of performance than their respective baseline levels (see Table 1, Figure 5). This finding further supports the non-statistically significant trend on digit span and suggests that both active TBS protocols may induce modulations of performance toward the same direction.

**Table 1:** Mean scores (*M*), standard deviations (*SD*), and medians (Mdn) obtained in the baseline (pre) and after stimulation (post) assessments.

		**Pre**									**Post**								
		**Sham**			**cTBS**			**iTBS**			**Sham**			**cTBS**			**iTBS**		
		***M***	***SD***	**Mdn**	***M***	***SD***	**Mdn**	***M***	***SD***	**Mdn**	***M***	***SD***	**Mdn**	***M***	***SD***	**Mdn**	***M***	***SD***	**Mdn**
3-back	Acc.	50.4	3.6	50.0	47.8	5.7	46.5	48.8	6.7	51.0	53.2	3.9	54.0	49.4	4.1	50.5	51.0	6.0	52.0
	RT (ms)	1042.7	282.2	963.5	1183.4	215.1	1232.5	1050.8	183.4	1079.1	891.5	274.7	811.4	1121.5	276.0	1162.9	958.2	179.8	979.7
Digit Backwards	Span	5.8	0.6	6.0	5.5	1.1	6.0	4.9	1.1	5.0	5.8	1.5	6.0	5.9	1.2	6.0	5.5	1.1	5.5
	Strings	8.1	1.7	8.0	7.8	2.2	7.5	6.5	1.7	7.0	8.4	2.4	8.5	8.8	2.4	9.0	7.9	2.0	8.0
Stroop	WR	116.4	12.2	116.5	115.3	9.4	116.0	109.3	9.8	106.5	121.1	17.3	120.0	120.7	11.8	118.5	113.2	11.5	112.0
	CN	75.5	12.1	75.0	72.7	9.5	73.0	72.6	11.9	71.5	82.6	10.5	81.5	75.5	9.1	75.5	75.6	9.7	75.0
	CW	52.4	10.1	47.5	50.2	8.3	51.0	44.7	8.0	43.0	58.9	12.2	54.5	53.9	7.6	55.5	50.8	8.0	49.5
	IS	6.8	7.0	6.1	5.8	5.3	7.3	1.1	5.6	1.8	9.9	9.1	8.6	7.7	4.7	8.2	5.5	4.9	5.7
Tower of Hanoi	Mov.	91.8	55.2	74.0	105.4	52.5	89.5	81.3	33.9	72.0	82.0	39.1	75.0	73.8	34.6	68.0	72.7	39.1	64.5
	Time (s)	356.9	268.4	275.5	381.3	325.9	283.5	274.5	161.3	208.5	197.8	91.5	191.5	202.2	139.0	159.5	186.1	129.3	150.5

*Acc., Accuracy, number of correct responses over 60 trials; RT, reaction times in milliseconds; Span, maximum string length; Strings, total number of strings correctly recalled; WR, Word Reading; CN, Color Naming; CW, Color-Word; IS, Interference Score; Mov., Number of movements to complete the task; Time., time to complete the task in seconds*.

### 3-back task

Following both sham (*Z* = −2.75; *p* = 0.006) and iTBS (*Z* = −2.28; *p* = 0.023) protocols there was a significant decrease in response reaction times for correct responses in the 3-back task, an effect that was not found following cTBS (*Z* = −1.33; *p* = 0.182). Regarding accuracy, sham stimulation showed a significant increase in the number of correct responses comparing scores pre vs. post stimulation (*Z* = −2.01; *p* = 0.045). This effect was not observed following cTBS (*Z* = −1.11; *p* = 0.265) nor iTBS (*Z* = −0.75; *p* = 0.454), suggesting that both active TBS conditions delivered to the left dlPFC prevented the appearance of practice effects, at least for accuracy in the iTBS protocol (Figure 5).

### Stroop test

Both cTBS (*Z* = −2.44; *p* = 0.015) and iTBS (*Z* = −2.40; *p* = 0.016) conditions showed significant increases of performance in the WR score of the Stroop Test, which were not found in the sham group (*Z* = −1.47; *p* = 0.142). This result indirectly suggests an impact of both cTBS and iTBS on Stroop WR but is unable to discriminate between these two active patterns.

For the CN score, sham (*Z* = −2.87; *p* = 0.004) and cTBS (*Z* = −2.61; *p* = 0.009) were the TMS modalities that, in contrast to iTBS (*Z* = −1.69; *p* = 0.091), significantly increased scores. The lack of performance increases with iTBS indirectly suggests an unexpected suppressive impact of this TBS pattern in the CN score of the Stroop task.

Regarding the CW score, our data revealed that the three stimulation modalities, sham (*Z* = −2.91, *p* = 0.004), cTBS (*Z* = −2.30, *p* = 0.021), and iTBS (*Z* = −2.36, *p* = 0.018) increased it. Taking into account the results in the WR and CN scores, this stimulation condition-independent effect is probably due to different mechanisms: an improved CN after sham stimulation, a general improvement after cTBS and a better interference control after iTBS (given the improvement in WR but not in CN).

Lastly, IS scores before and after TMS showed significant increases following sham stimulation (*Z* = −2.12; *p* = 0.034) and iTBS (*Z* = −2.04; *p* = 0.041), but not after cTBS pattern (*Z* = −1.41; *p* = 0.158). Similar to what we observed in the CW score, while the increased IS after sham might be caused by an improved CN due to practice, the lack of performance increases with cTBS could be explained by equivalent increases in the remaining scores (WR, CN, and CW). Regarding iTBS, an improved IS regardless of an increased WR and decreased CN indirectly suggests that iTBS pattern could have induced the expected positive behavioral impact on the IS score when delivered to the left dlPFC region (Figure 5).

### Tower of hanoi

No significant differences in the number of required movements were found comparing pre vs. post stimulation performance values following sham stimulation (*Z* = −0.31; *p* = 0.754), cTBS (*Z* = −1.14; *p* = 0.254), or iTBS (*Z* = −0.35; *p* = 0.724). This outcome suggests an absence of practice effect in this task and a null effect for both active TBS protocols.

Regarding the time needed to complete the task, participants under cTBS required significantly less time (*Z* = −2.20; *p* = 0.028) following stimulation, an effect which was not found either following iTBS (*Z* = −1.25; *p* = 0.209) or sham stimulation (*Z* = −1.73; *p* = 0.084). Regardless of the hypothesized cTBS suppressive effects on cortico-spinal excitability, this result supports the possibility that this protocol might have unexpectedly sped up performance (Figure 5).

## Discussion

Using a neuropsychological battery of well-established clinically relevant tasks, no statistically significant differences were found between the changes produced by the three stimulation conditions (iTBS, cTBS, and sham). This outcome suggests that stimulation modality (either active TBS vs. sham or iTBS vs. cTBS) had no bearing on behavioral processes in which the dlPFC is known to be critically involved when tested in healthy individuals with neuropsychological tasks used for clinical assessment. Interestingly, however, when pre-post stimulation differences were considered individually for each condition, active stimulation protocols applied (iTBS and cTBS) induced occasionally changes in participants' performance, which did reach statistical significance (see Figure 4).

In this sense, the presence or the absence of significant pre/post modulatory effects for active TMS conditions, different from those found after sham stimulation for some tests, is suggestive of a mild but significant task-dependent effect of focal TMS, hence deserving some attention. In the next sections, we will discuss pre vs. post differences in an attempt to understand the mechanisms underlying the unexpected subtle effects of stimulation and the causes of the absence of statistically significant differences between groups.

### Subtle but differential effects of TMS modalities compared to control condition

Improvement of performance after either iTBS or cTBS in those tasks that did not show practice effects in the control stimulation condition might be considered as an effect of the stimulation. Analogously, the absence of changes after stimulation in scores that did show practice effects in the control stimulation group could be considered a consequence of the changes in brain activity induced by the active stimulation. Nonetheless, any comparisons should be made with caution since “the difference between significant and not significant is not itself statistically significant” (Gelman and Stern, 2006; Nieuwenhuis et al., 2011). For this reason, we bear in mind the fact that the scores of the three groups at baseline did not show statistically significant differences and hence can be considered equivalents, and we will also take into account effect sizes (ES).

That being said, to better understand the potential subtle effects of stimulation, we first need to consider the presence or absence of significant changes observed in the control condition (sham TBS), which can only be interpreted as genuinely caused by the repetition of neuropsychological tests, i.e., by practice effects (Calamia et al., 2012). For the sham TBS, our data revealed significant pre vs. post improvements in the 3-back task (in both measures, accuracy and reaction time, ES 0.74 and 0.54, respectively) and also in the Stroop Test (CN, CW, and IS, ES 0.62, 0.58, and 0.38, respectively), similar to what has previously been reported elsewhere (Connor et al., 1988; Dulaney and Rogers, 1994; Sanchez-Carrion et al., 2008). In contrast, the Digit Backward (span and strings), Stroop WR score, and the Tower of Hanoi tests did not show any significant change following sham stimulation.

Now that practice effects observed in the sham condition have been described, we will focus on the differential impact of cTBS and iTBS on these same evaluation tasks. On the one hand, cTBS induced impaired performance in the 3-back task, in terms of both accuracy and reaction times, since the improvement due to practice, present after sham TBS, was absent in this group (ES 0.34 and 0.25, respectively). The same occurred with the IS of the Stroop Test (ES 0.37). Nevertheless, the cTBS group increased the number of series correctly recalled during the Digits Backward task (ES 0.4), increased the WR score in the Stroop Test (ES 0.51), and reduced the time taken to complete the Tower of Hanoi (ES 0.71), improvements that were not present after sham TBS. On the other hand, iTBS did not induce accuracy improvements for the 3-back task (ES 0.34), nor the CN score of the Stroop Test (ES 0.28), an effect that we did observe following sham TBS. Nevertheless, iTBS increased the number of series correctly recalled in the Digits Backward task (ES 0.76), and improved the WR score in the Stroop Test (ES 0.38).

### Possible mechanisms explaining the pre/post variations

A recent review suggests that the cognitive enhancement promoted by rTMS can be explained by three different mechanisms: (1) the direct modulation of the targeted cortical region; (2) the disruption of a competing processing or “addition-by-subtraction,” and (3) by nonspecific effects related, for example, to the clicking sound and tactile scalp sensations accompanying each pulse (see Luber and Lisanby, 2014 for detailed description).

*Direct modulation* refers to the up-regulation of excitability in the specific area needed for task performance. This mechanism could explain improvements in inhibitory control as measured by the Stroop Test mediated by iTBS. After iTBS, the increased WR score and the absence of an improvement in the CN score might have produced lower CW and IS scores. Nonetheless, both scores improved. Since, in CW condition, participants should inhibit the ongoing response of reading the word (facilitated after iTBS) in order to name the color (impaired after iTBS), lower scores were to be expected. On the other hand, for the IS score, the higher the number of words read (WR score), the lower the IS can be since it is calculated by subtracting from CW the product of WR and CN divided by WR plus CN. Therefore, considering global performance in the Stroop Test following iTBS, the inhibitory control capacity improved (iTBS ES 0.82). Similar increases in inhibitory control, revealed as faster responses to incongruent word-color conditions, have also been encountered after high-frequency rTMS over left dlPFC (Vanderhasselt et al., 2006; Kim et al., 2012). The left dlPFC is directly involved in inhibitory control processes (Miller and Cohen, 2001; Cipolotti et al., 2016), thus the increased excitability of this region can lead to a better performance. Nonetheless, in our study, the excitability decrease hypothetically caused by cTBS (Tupak et al., 2013) did not hinder the capacity for inhibitory control, unlike in a previous study (Lowe et al., 2014). This lack of cTBS effect might be due to compensatory processes between hemispheres already observed in right inferior frontal gyrus activity after cTBS over the contralateral area in a WM task (Lee and D'Esposito, 2012).

The second mechanism, *addition by subtraction*, consists in disrupting part of a network involved in a competing process with the task being performed, an effect resulting in temporary network reorganization. This mechanism could explain the pre- vs. post-TBS performance changes in the Tower of Hanoi, where cTBS significantly decreased the time to complete the task after stimulation (i.e., improved performance) and the ES were >0.71 in both time and movements. This “paradoxical facilitation” after decreasing cortical excitability could be explained by a disruption of a competing process localized in the left hemisphere, probably causing a transcallosal facilitation in the contralateral region. Indeed, different studies show the involvement of the right dlPFC in planning abilities, visuospatial memory, and/or response inhibition, the three main processes measured by the Tower of Hanoi (Epstein et al., 2002; Fincham et al., 2002; Sack et al., 2005; Srovnalova et al., 2012; Kaller et al., 2013; Fried et al., 2014; Cipolotti et al., 2016).

The third mechanism, the *non-specific effects*, refer to the effects not directly related to the stimulation *per se*, but to peripheral sensations (cross-modal sensory interactions) accompanying the delivery of TMS pulses. These effects are supposed to be present both in active and sham conditions, nevertheless, we found an effect of stimulation associated not to the specific pattern employed to modulate brain activity but to the cognitive load of the WM tasks. Our results show that both, the iTBS and cTBS protocols, produced similar outcomes on WM performance: they improved the Digits Backward score (with ES of 0.76 and 0.40, respectively compared to sham TMS, ES of 0.16), and, at the same time, they blocked the benefits of practice effects in the 3-back task (with an ES of 0.34 in both cases compared to 0.73 in sham). Thus, TBS protocols improved capacity to some extent (i.e., in Digits Backward) but, under high cognitive load conditions, such as the permanent updating of the stored items required in the 3-back task, the modulation of cortical excitability promoted by TBS might have hindered performance. The lack of such effects following sham TBS prevents us referring to these as “non-specific” and rather indicates an impact of patterned rTMS, independently of the specific temporal features of the stimulation protocol. In this case, the modulation of WM performance could be explained by the stochastic resonance phenomenon applied to stimulation, hypothesizing that the addition of some limited level of “noise” to a nonlinear system enhances information processing, whereas too much noise can interfere with processing (Moss et al., 2004). TMS has shown to have the ability to induce stochastic resonance effects, which might underlie some of its effects on perceptual cognition (Abrahamyan et al., 2011; Schwarzkopf et al., 2011). Moreover, the induction of the latter phenomenon has also been proposed as a plausible explanation for the effects driven by neuromodulation using transcranial electrical stimulation (Fertonani and Miniussi, 2016).

WM execution is thought to be based on dynamic coding, or moment to moment activity fluctuations in the prefrontal cortex (Stokes, 2015). General enhancements or impairments of WM processes after TBS could have been caused by the facilitation or the nuisance of this dynamic coding process underlying WM, highly sensitive to cognitive load. Two studies using TBS and the n-back task have reported results supporting this hypothesis. Hoy et al. (2016), showed improvements in the 2-back, but not in the 3-back task following iTBS over the left dlPFC. On the contrary, using cTBS over the same area, Schicktanz et al. (2015) found an impairment in the 2-back and no effect on the 3-back. Performance differences for high vs. low WM load tested across n-back task conditions (from 0- to 3-back) could not be verified on a recent meta-analysis of WM improvements driven by non-invasive brain stimulation delivered over the dlPFC, in which all task conditions were analyzed together (Brunoni and Vanderhasselt, 2014). Outcomes in the Digits Backward test cannot be easily compared to previous studies using TBS, since to the best of our knowledge, only two studies have used this task as an outcome measure after rTMS, and the stimulation protocols used in both differ greatly from ours (Rami et al., 2003; Aleman and van't Wout, 2008). We abstained from considering studies using the Digits Forward or a combined score integrating the Digits Forward and Backward tests reported in the WAIS, since forward and backward recall of digits involve different cognitive processes, which do not rely on the same neural systems (Reynolds, 1997; Gerton et al., 2004).

Prior studies using TBS protocols in non-motor areas also argue in favor of rather unspecific TBS effects. Hoy et al. (2016) suggest that iTBS may improve the balance between excitatory and inhibitory inputs allowing better information transmission. Gratton et al. (2013) show that cTBS applied over regions associated with cognitive control promotes a general, widespread and non-specific increase of functional connectivity across frontal, parietal, and cingulate regions, compared with more specific connectivity changes after stimulating the primary sensory cortex. Finally, Woźniak-Kwaśniewska et al. (2014), using offline scalp EEG recordings, reported similar after-effects following either high-frequency rTMS (10 Hz), low-frequency rTMS (1 Hz), and also cTBS or iTBS delivered over the left dlPFC.

To our knowledge, only one study has previously compared changes in cognitive performance following the delivery of cTBS vs. iTBS over the left dlPFC (Grossheinrich et al., 2009). These authors administered different tests to assess EF and WM, together with verbal memory and attention. The only relevant finding concerning cognitive performance was a non-significant trend showing a difference on the Letter-Number sequencing task from the WAIS (a WM task) when comparing performance following iTBS vs. cTBS (ES 0.35), and iTBS vs. sham (ES 0.25). When all tests were considered together as a single measure, authors reported worse performance after iTBS as compared with sham. Additionally, iTBS, instead of displaying a general excitatory effect (as recorded in primary motor areas), was noted as modulating neural networks “in a more complex manner”. The lack of studies comparing both TBS protocols makes it difficult to provide additional support to the different mechanisms hypothesized here to explain our mixed outcomes.

### Why only subtle effects

Two main sets of apparently “conflicting” results emerge from the analyses of the current study, on the one hand, the absence of significant differences between conditions, and on the other hand, the presence of pre-post stimulation differences within conditions. On such basis, a twofold conclusion can be derived. First, no straightforward assumptions about TMS effects (TBS protocols in our case) as probed in the primary motor cortex (with MEPs) can be taken and extrapolated to the behavioral impact of these same patterns when applied to other cortical regions (in our case the dlPFC). Second, in order to better gauge the impact of different TMS protocols on the dlPFC and their effects on cognitive performance, a close and detailed analysis of more subtle effects is compulsory. Different reasons might explain why we did not find statistically significant differences across the three TMS conditions (iTBS, cTBS, sham) and they are probably related to the characteristics of stimulation protocols and their ability to modulate the target area. Nonetheless, study limitations related to the design, the sample characteristics, the statistical analyses and the tasks employed cannot be underestimated.

The two stimulation protocols selected in this study, iTBS and cTBS, applied over the primary motor cortex have consistently shown long term opposite changes in the amplitude of motor evoked potentials (Wischnewski and Schutter, 2015). However, the cortical excitability of the motor cortex and the dlPFC shows fundamental differences (Farzan et al., 2009), and so do their connectivity patterns. It should not be forgotten that TMS, and specifically TBS patterns, affect not only the cortical targeted area directly receiving the stimulation but also a network of other areas functionally and structurally connected to it (e.g., Reithler et al., 2011; Gratton et al., 2013). Thus, given the role of the dlPFC as cortical hub responsible for the transmission and integration of information between widespread functional networks (Cole et al., 2012; van den Heuvel and Sporns, 2013), it should not be surprising that changes in excitability caused by TBS over this area may have diverse effects on cognitive performance.

To our knowledge, only two studies have compared the electrophysiological changes produced by iTBS and cTBS over the dlPFC (Grossheinrich et al., 2009; Wischnewski and Schutter, 2015). Grossheinrich et al. (2009), measured resting state EEG and only found changes in the α2 band after iTBS but not after cTBS or sham stimulation. On the contrary, Wischnewski and Schutter (2015), compared the spectral EEG power of pre- vs. post-TMS and found a decrease of delta and theta power on left prefrontal areas similar for all active stimulation conditions. These discrepancies support the idea of complex and yet to be explored modulation of the cortical activity promoted by TBS over the dlPFC and its associate networks.

State-dependency effects observed in stimulation studies (Silvanto and Pascual-Leone, 2008) need to be also seriously considered as a potential confound. Those effects refer to changes of stimulation impact driven by TMS on physiological or behavioral outcomes, as influenced by the ongoing state of activity of the distinct neural populations within the targeted cortical region during the delivery of the pulses. Regional activity state can be modulated with either stimulus presented or cognitive activities performed by the participant immediately prior during or even immediately following the stimulation, modifying the magnitude and eventually the sense of the TMS driven excitability hence impacting its derived behavioral performance changes (e.g., Silvanto et al., 2007; Li et al., 2016). In our study the same procedure prior, during and following stimulation sessions was followed throughout participants. Nonetheless, the impact of TBS could have been influenced by differences in ongoing regional activity operating on each participant. We opted not to ask participants to perform a control task during stimulation delivery (and by doing so control better activity state) because unlike long classical TMS patterns, TBS stimulation is very short (at most ~40 s), and to avoid any confounding effect of a task performance (which would likely have involved the dlPFC differentially across participants). Hence, differences in specific cognitive activity carried out by participants during stimulation could have introduced some variability. For that reason, future studies should address TMS-brain interaction effects in order to draw more accurate conclusions about the capabilities of TBS for modulating cognitive performance, specially when used as a rehabilitation tool in clinical populations. In this context, the engagement of the participants in a specific task during the delivery of stimulation has the potential to reduce interindividual variability and most importantly, eventually, increase the magnitude and specificity of the TMS effects (Romei et al., 2016).

Some additional limitations of the present study are also worth noting. First, we used a rather small sample size and implemented a study design based on independent cohorts tested in parallel, associated each one with a single stimulation condition. This design does not allow internal comparisons of performance across active and sham TBS conditions in the same population of participants. Unfortunately, repetition of tasks such as ours showing strong test-retest learning effects (hence very sensitive to intra-session and inter-session learning), would have made it very difficult to disentangle the effects of practice from those induced by the stimulation, therefore a crossover design was excluded early on. Our sample size was based on a priori calculation for planned comparisons (i.e., repeated measures ANOVA) to achieve a statistical power of 0.8 and assuming a medium effect size (0.5), based on previous studies that show larger effect sizes after applying TBS compared to rTMS (Lowe et al., 2014; Hoy et al., 2016). Unfortunately, the non-normal distribution of our datasets, forced us to perform nonparametric tests, which led to lower statistical power, and risk for type II errors. Nevertheless, the exact tests performed and the high p values found in most cases make this possibility rather unlikely. Although, as a general recommendation, large sample sizes are always desirable to be able to confirm or rule out the effects of stimulation, even with 12 participants per group we could extract some relevant conclusions about the effects of the different TBS protocols over the dlPFC.

Regarding the characteristics of our cohorts, the outcomes we here report for healthy participants cannot be easily compared with the ones reported in patients (Bermpohl et al., 2006). Improvement of performance in healthy participant tends to show ceiling effects and, in general, smaller effect sizes compared with patients (Brunoni and Vanderhasselt, 2014), who presumably suffer disorders in the excitatory/inhibitory balance and local brain synchrony (Yizhar et al., 2011). The limited behavioral responses observed in healthy subjects, such as the ones participating in our study, have also been related to fast-acting compensatory/reorganization plastic mechanisms (Thut and Pascual-Leone, 2010). These processes can be especially relevant in offline paradigms, for which the stimulation is delivered prior to task performance, so changes in brain excitability directed at preserving its *status quo* can rapidly counteract region excitability/activity modulations, particularly in an intact healthy brain (Lee et al., 2003).

Another limitation might be related with the interindividual anatomical and functional differences. Although, cortical target selection was based on previous studies showing the engagement of dlPFC in WM processes and adapted to fit the anatomy of each individual participant, the specific site contributing to a given cognitive process may differ due to interindividual differences in cortical folding and connectivity (Fischl et al., 2008; Mennes et al., 2010).

Finally, at odds with finely tuned, computer-based tasks targeting as purely as possible the contributions of well-restricted cortical regions or networks frequently employed in TMS and fMRI paradigms, the battery of neuropsychological tasks employed in the current study was more likely to recruit widespread neural networks in addition to the dlPFC (e.g., Peterson et al., 2002; Owen et al., 2005), hence invoking a large variety of resources. Moreover, the impact of TBS over dlPFC might be too mild to modulate performance in ecological and holistic tasks that cannot be easily titrated in difficulty (to avoid ceiling or floor effects), and requiring a massive change in excitability or metabolism, in a large area within the dlPFC, in order to either impair (cTBS) or facilitate (iTBS) behaviors.

## Conclusions

Studies gauging the modulatory effects of TBS protocols with neuropsychological tasks often used for clinical assessment could prove particularly relevant for the neurostimulation field, given their greater ecological validity in clinical practice, compared to finely titrated but poorly ecological experimental paradigms (Pascual-Leone et al., 2012). In this context, our results relay useful knowledge about the impact of patterned TMS (iTBS and cTBS) over the left dlPFC, which is one of the most extensively explored areas of the human brain, and a common target for non-invasive neurostimulation in attempts to explore, enhance or restore cognition (Pascual-Leone et al., 2012; Anderkova and Rektorova, 2014). We failed to find TMS-pattern specific differences on a battery of prefrontal tasks performed under the influence of iTBS, cTBS, and sham TBS. Nonetheless, we report a series of significant task- and outcome measure-dependent changes comparing performance prior vs. following stimulation which did differ according to the stimulation protocol. Taken as a whole, the interpretation of our outcomes indirectly suggest that cTBS and iTBS protocols combined with complex clinical tasks, do not share the same features delivered on higher-order hub areas (such as the dlPFC) as compared to on a primary motor, or primary sensory (visual) regions in which they have shown to either increase (iTBS) or decrease (cTBS) excitability. Our mixed results could also reflect the existence of different mechanisms of TMS on cognitive performance, revealing, for example, an impact on WM which is unrelated to stimulation protocol, a direct modulation of interference control, and an indirect or “addition-by-subtraction” effects over spatial planning abilities. Given the differential effects of TBS over the dlPFC compared with M1 (Woźniak-Kwaśniewska et al., 2014), additional research on this protocol, both at the neuropsychological and electrophysiological level, is needed to fully understand its modulatory capacity on cognitive processing and the interactions between the TMS pulses and the participant's brain state at the time of stimulation. Overall, the final take-home message is that the impact of a given TBS protocol (and this can be extended to any conventional TMS patterns), has to be thoroughly evaluated on the specific cortical target by means of clinically relevant tasks before it can be effectively used for diagnosis or treatment. Hence, assuming that TBS protocols will show equal modulatory power and direction independently on the cortical region, behavioral evaluation measures, or population of participants, is the perfect receipt for frustration.

## Author contributions

EM conceived the study and participated in the design of the experimental sessions with DR, RV, and AV. RV and MP performed the experiments and acquired the data. MB, RV, and DR performed the statistical analyses. RV, EM, and AV contributed to the interpretation of the data. RV drafted the work. DR, EM, AV, MB, and MP contributed to critically revise and rewrite its intellectual content. All authors gave the final approval to the manuscript and agree to be accountable for all aspects of the work.

### Conflict of interest statement

The authors declare that the research was conducted in the absence of any commercial or financial relationships that could be construed as a potential conflict of interest. The reviewer ER and handling Editor declared their shared affiliation, and the handling Editor states that the process nevertheless met the standards of a fair and objective review.
